# 3′,6′-Bis(diethyl­amino)-2-phenyl­spiro[isoindoline-1,9′-xanthen]-3-one

**DOI:** 10.1107/S1600536809020248

**Published:** 2009-06-06

**Authors:** Wu-Jian Deng, Di Sun, Bing-Yuan Su, Shu-Ping Wang, Hong Zheng

**Affiliations:** a Key Laboratory of Analytical Sciences, Ministry of Education, Department of Chemistry, College of Chemistry and Chemical Engineering, Xiamen University, Xiamen 361005, People’s Republic of China; b Department of Chemistry, Xiamen University, Xiamen 361005, People’s Republic of China

## Abstract

The title compound, C_34_H_35_O_2_N_3_, was synthesized by the reaction of 2-[3,6-bis­(diethyl­amino)-9*H*-xanthen-9-yl]benzoyl chloride with aniline. In the mol­ecular structure, the dihedral angles between the isoindoline and xanthene planes and between the isoindoline and benzene planes are 86.9 (3) and 47.0 (2)°, respectively. The mol­ecular packing in the crystal structure is stabilized by weak C—H⋯O hydrogen bonding.

## Related literature

For applications of rhodamine-based dyes as probes and sensors, see: Zheng *et al.* (2008 ▶); Wu *et al.* (2007 ▶). For a related structure, see: Kwon *et al.* (2005 ▶).
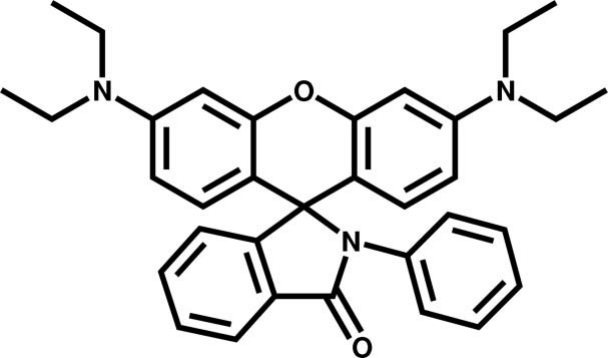


## Experimental

### 

#### Crystal data

C_34_H_35_N_3_O_2_
*M* *_r_* = 517.65
Monoclinic, 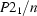

*a* = 12.0213 (5) Å
*b* = 12.6315 (4) Å
*c* = 18.9700 (7) Å
β = 107.456 (4)°
*V* = 2747.88 (18) Å^3^
*Z* = 4
Mo *K*α radiation
μ = 0.08 mm^−1^
*T* = 173 K
0.30 × 0.20 × 0.20 mm

#### Data collection

Oxford Diffraction Xcalibur diffractometer with a Sapphire3 (Gemini Ultra Mo) detector
Absorption correction: none
29794 measured reflections
5403 independent reflections
4396 reflections with *I* > 2σ(*I*)
*R*_int_ = 0.025

#### Refinement

*R*[*F* ^2^ > 2σ(*F* ^2^)] = 0.040
*wR*(*F* ^2^) = 0.103
*S* = 1.06
5403 reflections
352 parameters
H-atom parameters constrained
Δρ_max_ = 0.22 e Å^−3^
Δρ_min_ = −0.19 e Å^−3^

### 

Data collection: *CrysAlis CCD* (Oxford Diffraction, 2007 ▶); cell refinement: *CrysAlis RED* (Oxford Diffraction, 2007 ▶); data reduction: *CrysAlis RED*; program(s) used to solve structure: *SHELXS97* (Sheldrick, 2008 ▶); program(s) used to refine structure: *SHELXL97* (Sheldrick, 2008 ▶); molecular graphics: *ORTEP-3* (Farrugia, 1997 ▶); software used to prepare material for publication: *SHELXL97*.

## Supplementary Material

Crystal structure: contains datablocks I, global. DOI: 10.1107/S1600536809020248/xu2519sup1.cif

Structure factors: contains datablocks I. DOI: 10.1107/S1600536809020248/xu2519Isup2.hkl

Additional supplementary materials:  crystallographic information; 3D view; checkCIF report

## Figures and Tables

**Table 1:** Hydrogen-bond geometry (Å, °)

*D*—H⋯*A*	*D*—H	H⋯*A*	*D*⋯*A*	*D*—H⋯*A*
C31—H31*B*⋯O2^i^	0.99	2.56	3.4032 (19)	144

Symmetry code: (i) 
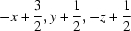
.

**Fig. 1.**
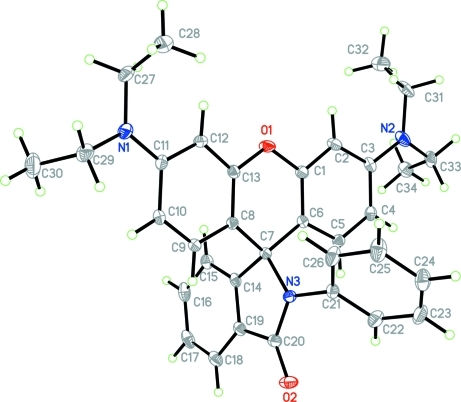
The molecular structure of the title compound with 30% probability displacement ellipsoids for non-H atoms.

## supplementary crystallographic information

### Comment

Rhodamine-based dyes, known by their excellent spectroscopic properties of large molar extinction coefficient and high fluorescence quantum yield (Wu *et al.*, 2007), have found applications in the study of complex biological systems and environmental analysis as molecular probes. In the present paper, the structure of title compound has been determined as part of a research program involving the synthesis and nitric oxide sensing (Zheng *et al.*, 2008).

The molecular structure is depicted in Fig. 1. Bond lengths and angles are in good agreement with previous reported for similar compounds (Kwon *et al.*, 2005). The dihedral angle between isoindoline and xanthene mean planes is 86.9 (3)° The dihedral angle between the isoindoline and benzene ring mean planes is 47.0 (2)°. Weak C—H···O hydrogen bonding (Table 1) helps to stabilize the crystal structure.

### Experimental

To a solution of 3',6'-bis(diethylamino)-3*H*-spiro[isobenzofuran-1,9'- xanthen]-3-one (1.3 g, 2.8 mmol) in dry 1,2-dichloroethane (10.0 ml) at room temperature, phosphorus oxychloride (1.4 g, 8.4 mmol) was added dropwise over a period of 5 min. After being refluxed for 4 h, the reaction mixture was cooled and concentrated under vacuum to give 2-(3,6-bis(diethylamino)-9*H*-xanthen-9-yl)benzoyl chloride. The chloride salt was dissolved in dry acetonitrile (12.0 ml). This solution was added dropwise to a solution of aniline (1.6 g, 17.5 mmol) in dry acetonitrile (7.5 ml) containing triethylamine (10.0 ml). After stirring for 4 h at room temperature, the mixture was concentrated under vacuum and the crude product was purified by column chromatography (ethyl acetate/dichloromethane, 1:20) to give the title compound as a white solid in 72% yield. Single crystals of the title compound were obtained by slow evaporation of a dichloromethane/methanol solution (5:1 v/v). The product was analyzed by atmospheric-Pressure Chemical Ionization (APCI) mass spectrometry (positive mode). The molecular peak appeared at a mass/charge ratio of 518.5.

### Refinement

H atoms were placed geometrically with C—H = 0.95 (aromatic), 0.98 (methyl) and 0.99 Å (methylene), and refined using a riding atom model with U_iso_(H) = 1.5U_eq_(C) for methyl and 1.2U_eq_(C) for the others.

### Crystal data

**Table:**

C_34_H_35_N_3_O_2_	*F*(000) = 1104
*M**_r_* = 517.65	*D*_x_ = 1.251 Mg m^−^^3^
Monoclinic, *P*2_1_/*n*	Mo *K*α radiation, λ = 0.71073 Å
Hall symbol: -P 2yn	Cell parameters from 16600 reflections
*a* = 12.0213 (5) Å	θ = 2.4–32.7°
*b* = 12.6315 (4) Å	µ = 0.08 mm^−^^1^
*c* = 18.9700 (7) Å	*T* = 173 K
β = 107.456 (4)°	Block, colourless
*V* = 2747.88 (18) Å^3^	0.30 × 0.20 × 0.20 mm
*Z* = 4	

### Data collection

**Table:**

Oxford Diffraction Xcalibur diffractometer with a Sapphire3 (Gemini ultra Mo) detector	4396 reflections with *I* > 2σ(*I*)
Radiation source: fine-focus sealed tube	*R*_int_ = 0.025
graphite	θ_max_ = 26.0°, θ_min_ = 2.4°
Detector resolution: 16.1903 pixels mm^-1^	*h* = −14→14
φ and ω scans	*k* = −15→15
29794 measured reflections	*l* = −21→23
5403 independent reflections	

### Refinement

**Table:**

Refinement on *F*^2^	Primary atom site location: structure-invariant direct methods
Least-squares matrix: full	Secondary atom site location: difference Fourier map
*R*[*F*^2^ > 2σ(*F*^2^)] = 0.040	Hydrogen site location: inferred from neighbouring sites
*wR*(*F*^2^) = 0.103	H-atom parameters constrained
*S* = 1.06	*w* = 1/[σ^2^(*F*_o_^2^) + (0.0572*P*)^2^ + 0.4462*P*] where *P* = (*F*_o_^2^ + 2*F*_c_^2^)/3
5403 reflections	(Δ/σ)_max_ = 0.006
352 parameters	Δρ_max_ = 0.22 e Å^−^^3^
0 restraints	Δρ_min_ = −0.19 e Å^−^^3^

### Special details

**Table:**

Geometry. All e.s.d.'s (except the e.s.d. in the dihedral angle between two l.s. planes) are estimated using the full covariance matrix. The cell e.s.d.'s are taken into account individually in the estimation of e.s.d.'s in distances, angles and torsion angles; correlations between e.s.d.'s in cell parameters are only used when they are defined by crystal symmetry. An approximate (isotropic) treatment of cell e.s.d.'s is used for estimating e.s.d.'s involving l.s. planes.
Refinement. Refinement of *F*^2^ against ALL reflections. The weighted *R*-factor *wR* and goodness of fit *S* are based on *F*^2^, conventional *R*-factors *R* are based on *F*, with *F* set to zero for negative *F*^2^. The threshold expression of *F*^2^ > σ(*F*^2^) is used only for calculating *R*-factors(gt) *etc*. and is not relevant to the choice of reflections for refinement. *R*-factors based on *F*^2^ are statistically about twice as large as those based on *F*, and *R*- factors based on all data will be even larger.

### Fractional atomic coordinates and isotropic or equivalent isotropic displacement parameters (Å^2^)

**Table:**

	*x*	*y*	*z*	*U*_iso_*/*U*_eq_	
C1	0.71114 (11)	0.71818 (10)	0.15020 (7)	0.0228 (3)	
C2	0.82352 (12)	0.71273 (11)	0.14514 (8)	0.0267 (3)	
H2A	0.8558	0.7722	0.1277	0.032*	
C3	0.88993 (11)	0.62080 (11)	0.16541 (7)	0.0247 (3)	
C4	0.83537 (12)	0.53371 (10)	0.18815 (7)	0.0240 (3)	
H4A	0.8767	0.4690	0.2011	0.029*	
C5	0.72318 (11)	0.54138 (10)	0.19186 (7)	0.0219 (3)	
H5A	0.6893	0.4815	0.2077	0.026*	
C6	0.65727 (11)	0.63372 (10)	0.17327 (7)	0.0192 (3)	
C7	0.53398 (11)	0.64059 (10)	0.17788 (7)	0.0192 (3)	
C8	0.48969 (11)	0.75350 (9)	0.16449 (7)	0.0192 (3)	
C9	0.38198 (11)	0.78341 (10)	0.17203 (7)	0.0212 (3)	
H9A	0.3381	0.7321	0.1890	0.025*	
C10	0.33646 (11)	0.88340 (10)	0.15613 (7)	0.0225 (3)	
H10A	0.2624	0.8995	0.1617	0.027*	
C11	0.39947 (11)	0.96252 (10)	0.13143 (7)	0.0219 (3)	
C12	0.50750 (12)	0.93349 (10)	0.12374 (7)	0.0235 (3)	
H12A	0.5524	0.9844	0.1073	0.028*	
C13	0.54985 (11)	0.83143 (10)	0.13973 (7)	0.0214 (3)	
C14	0.44953 (11)	0.56635 (10)	0.12430 (7)	0.0214 (3)	
C15	0.41783 (12)	0.56501 (11)	0.04796 (8)	0.0275 (3)	
H15A	0.4507	0.6137	0.0216	0.033*	
C16	0.33642 (13)	0.49019 (12)	0.01089 (8)	0.0340 (4)	
H16A	0.3143	0.4871	−0.0415	0.041*	
C17	0.28681 (13)	0.41981 (12)	0.04928 (9)	0.0349 (4)	
H17A	0.2307	0.3698	0.0228	0.042*	
C18	0.31836 (12)	0.42201 (11)	0.12532 (9)	0.0308 (3)	
H18A	0.2847	0.3743	0.1519	0.037*	
C19	0.40063 (11)	0.49595 (10)	0.16190 (8)	0.0235 (3)	
C20	0.45099 (12)	0.51313 (10)	0.24234 (8)	0.0237 (3)	
C21	0.60912 (11)	0.62493 (10)	0.31955 (7)	0.0219 (3)	
C22	0.66767 (14)	0.54632 (12)	0.36746 (8)	0.0341 (3)	
H22A	0.6536	0.4739	0.3542	0.041*	
C23	0.74656 (14)	0.57334 (13)	0.43449 (8)	0.0393 (4)	
H23A	0.7852	0.5192	0.4676	0.047*	
C24	0.76971 (14)	0.67813 (13)	0.45384 (8)	0.0385 (4)	
H24A	0.8240	0.6965	0.4999	0.046*	
C25	0.71330 (15)	0.75539 (13)	0.40563 (9)	0.0439 (4)	
H25A	0.7297	0.8277	0.4182	0.053*	
C26	0.63245 (14)	0.72939 (11)	0.33859 (8)	0.0360 (4)	
H26A	0.5933	0.7838	0.3059	0.043*	
C27	0.41811 (13)	1.14312 (11)	0.08587 (8)	0.0327 (3)	
H27A	0.3609	1.1947	0.0563	0.039*	
H27B	0.4559	1.1081	0.0523	0.039*	
C28	0.51015 (15)	1.20246 (12)	0.14500 (10)	0.0418 (4)	
H28A	0.5487	1.2541	0.1216	0.063*	
H28B	0.5681	1.1522	0.1740	0.063*	
H28C	0.4732	1.2394	0.1776	0.063*	
C29	0.25151 (14)	1.09900 (12)	0.13220 (9)	0.0372 (4)	
H29A	0.2611	1.1744	0.1470	0.045*	
H29B	0.2431	1.0579	0.1747	0.045*	
C30	0.14156 (16)	1.08697 (19)	0.06812 (11)	0.0630 (6)	
H30A	0.0747	1.1124	0.0827	0.094*	
H30B	0.1302	1.0122	0.0540	0.094*	
H30C	0.1485	1.1285	0.0260	0.094*	
C31	1.06594 (13)	0.70793 (12)	0.15060 (10)	0.0379 (4)	
H31A	1.1479	0.7048	0.1825	0.045*	
H31B	1.0301	0.7720	0.1646	0.045*	
C32	1.06523 (17)	0.71910 (15)	0.07113 (10)	0.0540 (5)	
H32A	1.1085	0.7829	0.0659	0.081*	
H32B	0.9846	0.7248	0.0391	0.081*	
H32C	1.1022	0.6569	0.0569	0.081*	
C33	1.06374 (13)	0.51390 (12)	0.17186 (8)	0.0319 (3)	
H33A	1.0513	0.4745	0.2140	0.038*	
H33B	1.1485	0.5266	0.1831	0.038*	
C34	1.02314 (13)	0.44583 (12)	0.10308 (9)	0.0364 (4)	
H34A	1.0664	0.3789	0.1113	0.055*	
H34B	1.0372	0.4833	0.0613	0.055*	
H34C	0.9396	0.4313	0.0922	0.055*	
N1	0.35614 (10)	1.06343 (9)	0.11526 (7)	0.0306 (3)	
N2	1.00455 (10)	0.61526 (10)	0.16542 (7)	0.0332 (3)	
N3	0.52732 (9)	0.59642 (8)	0.25021 (6)	0.0205 (2)	
O1	0.65733 (8)	0.81453 (7)	0.12950 (6)	0.0307 (2)	
O2	0.42961 (9)	0.46357 (8)	0.29198 (6)	0.0345 (3)	

### Atomic displacement parameters (Å^2^)

**Table:**

	*U*^11^	*U*^22^	*U*^33^	*U*^12^	*U*^13^	*U*^23^
C1	0.0214 (7)	0.0196 (6)	0.0281 (7)	0.0008 (5)	0.0084 (6)	−0.0007 (5)
C2	0.0222 (7)	0.0232 (7)	0.0372 (8)	−0.0038 (5)	0.0128 (6)	−0.0019 (6)
C3	0.0176 (7)	0.0295 (7)	0.0257 (7)	0.0001 (5)	0.0045 (5)	−0.0075 (6)
C4	0.0218 (7)	0.0243 (7)	0.0244 (7)	0.0057 (5)	0.0048 (5)	−0.0009 (5)
C5	0.0229 (7)	0.0223 (7)	0.0203 (6)	−0.0001 (5)	0.0064 (5)	0.0006 (5)
C6	0.0169 (6)	0.0216 (6)	0.0188 (6)	−0.0003 (5)	0.0048 (5)	−0.0016 (5)
C7	0.0177 (6)	0.0211 (6)	0.0187 (6)	−0.0007 (5)	0.0053 (5)	0.0013 (5)
C8	0.0180 (6)	0.0198 (6)	0.0188 (6)	−0.0001 (5)	0.0040 (5)	−0.0001 (5)
C9	0.0185 (7)	0.0229 (7)	0.0221 (6)	−0.0016 (5)	0.0060 (5)	0.0013 (5)
C10	0.0169 (6)	0.0252 (7)	0.0241 (6)	0.0025 (5)	0.0041 (5)	−0.0009 (5)
C11	0.0217 (7)	0.0204 (6)	0.0207 (6)	0.0029 (5)	0.0019 (5)	0.0002 (5)
C12	0.0231 (7)	0.0206 (7)	0.0275 (7)	−0.0015 (5)	0.0085 (6)	0.0036 (5)
C13	0.0180 (7)	0.0229 (7)	0.0232 (6)	0.0007 (5)	0.0062 (5)	0.0001 (5)
C14	0.0153 (6)	0.0205 (6)	0.0273 (7)	0.0032 (5)	0.0049 (5)	−0.0040 (5)
C15	0.0227 (7)	0.0306 (7)	0.0285 (7)	0.0033 (6)	0.0068 (6)	−0.0008 (6)
C16	0.0259 (8)	0.0414 (9)	0.0286 (7)	0.0081 (6)	−0.0012 (6)	−0.0116 (6)
C17	0.0207 (7)	0.0287 (8)	0.0492 (9)	−0.0006 (6)	0.0015 (7)	−0.0164 (7)
C18	0.0219 (7)	0.0220 (7)	0.0483 (9)	0.0001 (6)	0.0100 (6)	−0.0046 (6)
C19	0.0181 (7)	0.0194 (6)	0.0334 (7)	0.0035 (5)	0.0083 (6)	−0.0026 (5)
C20	0.0217 (7)	0.0186 (6)	0.0341 (7)	0.0024 (5)	0.0132 (6)	0.0022 (5)
C21	0.0197 (7)	0.0269 (7)	0.0197 (6)	0.0037 (5)	0.0071 (5)	−0.0011 (5)
C22	0.0398 (9)	0.0287 (8)	0.0311 (8)	0.0039 (7)	0.0065 (7)	0.0052 (6)
C23	0.0395 (9)	0.0459 (10)	0.0277 (8)	0.0082 (7)	0.0029 (7)	0.0120 (7)
C24	0.0360 (9)	0.0513 (10)	0.0233 (7)	0.0056 (7)	0.0014 (6)	−0.0050 (7)
C25	0.0471 (10)	0.0341 (9)	0.0377 (9)	0.0041 (7)	−0.0069 (8)	−0.0114 (7)
C26	0.0410 (9)	0.0259 (7)	0.0318 (8)	0.0069 (6)	−0.0033 (7)	0.0000 (6)
C27	0.0348 (8)	0.0229 (7)	0.0407 (8)	0.0070 (6)	0.0118 (7)	0.0098 (6)
C28	0.0411 (10)	0.0309 (8)	0.0529 (10)	0.0000 (7)	0.0131 (8)	0.0054 (7)
C29	0.0386 (9)	0.0259 (7)	0.0519 (10)	0.0125 (7)	0.0209 (8)	0.0079 (7)
C30	0.0363 (10)	0.0903 (16)	0.0623 (12)	0.0247 (10)	0.0148 (9)	0.0299 (11)
C31	0.0181 (7)	0.0366 (9)	0.0593 (11)	−0.0060 (6)	0.0123 (7)	−0.0130 (7)
C32	0.0554 (12)	0.0458 (10)	0.0569 (11)	−0.0162 (9)	0.0110 (9)	0.0081 (9)
C33	0.0193 (7)	0.0420 (9)	0.0339 (8)	0.0091 (6)	0.0075 (6)	0.0021 (6)
C34	0.0303 (8)	0.0384 (8)	0.0430 (9)	0.0028 (7)	0.0149 (7)	−0.0043 (7)
N1	0.0279 (7)	0.0227 (6)	0.0424 (7)	0.0074 (5)	0.0123 (6)	0.0073 (5)
N2	0.0184 (6)	0.0320 (7)	0.0504 (8)	0.0006 (5)	0.0123 (6)	−0.0056 (6)
N3	0.0212 (6)	0.0196 (5)	0.0215 (5)	−0.0002 (4)	0.0075 (4)	0.0022 (4)
O1	0.0242 (5)	0.0210 (5)	0.0540 (7)	0.0036 (4)	0.0225 (5)	0.0089 (4)
O2	0.0385 (6)	0.0315 (6)	0.0391 (6)	−0.0055 (5)	0.0202 (5)	0.0072 (4)

### Geometric parameters (Å, °)

**Table:**

C1—O1	1.3789 (15)	C21—C22	1.3870 (19)
C1—C2	1.3846 (19)	C21—N3	1.4320 (16)
C1—C6	1.3854 (18)	C22—C23	1.382 (2)
C2—C3	1.3961 (19)	C22—H22A	0.9500
C2—H2A	0.9500	C23—C24	1.380 (2)
C3—N2	1.3796 (18)	C23—H23A	0.9500
C3—C4	1.413 (2)	C24—C25	1.370 (2)
C4—C5	1.3749 (19)	C24—H24A	0.9500
C4—H4A	0.9500	C25—C26	1.389 (2)
C5—C6	1.3945 (18)	C25—H25A	0.9500
C5—H5A	0.9500	C26—H26A	0.9500
C6—C7	1.5131 (18)	C27—N1	1.4588 (19)
C7—N3	1.5054 (16)	C27—C28	1.516 (2)
C7—C8	1.5170 (17)	C27—H27A	0.9900
C7—C14	1.5245 (17)	C27—H27B	0.9900
C8—C13	1.3842 (18)	C28—H28A	0.9800
C8—C9	1.3967 (18)	C28—H28B	0.9800
C9—C10	1.3735 (18)	C28—H28C	0.9800
C9—H9A	0.9500	C29—N1	1.4593 (19)
C10—C11	1.4161 (19)	C29—C30	1.511 (3)
C10—H10A	0.9500	C29—H29A	0.9900
C11—N1	1.3763 (17)	C29—H29B	0.9900
C11—C12	1.3985 (19)	C30—H30A	0.9800
C12—C13	1.3858 (18)	C30—H30B	0.9800
C12—H12A	0.9500	C30—H30C	0.9800
C13—O1	1.3795 (16)	C31—N2	1.4555 (19)
C14—C19	1.3773 (19)	C31—C32	1.512 (3)
C14—C15	1.3826 (19)	C31—H31A	0.9900
C15—C16	1.390 (2)	C31—H31B	0.9900
C15—H15A	0.9500	C32—H32A	0.9800
C16—C17	1.392 (2)	C32—H32B	0.9800
C16—H16A	0.9500	C32—H32C	0.9800
C17—C18	1.377 (2)	C33—N2	1.4520 (18)
C17—H17A	0.9500	C33—C34	1.516 (2)
C18—C19	1.3858 (19)	C33—H33A	0.9900
C18—H18A	0.9500	C33—H33B	0.9900
C19—C20	1.4790 (19)	C34—H34A	0.9800
C20—O2	1.2207 (16)	C34—H34B	0.9800
C20—N3	1.3744 (17)	C34—H34C	0.9800
C21—C26	1.3744 (19)		
			
O1—C1—C2	114.10 (11)	C24—C23—H23A	119.7
O1—C1—C6	123.08 (12)	C22—C23—H23A	119.7
C2—C1—C6	122.81 (12)	C25—C24—C23	119.08 (14)
C1—C2—C3	120.76 (12)	C25—C24—H24A	120.5
C1—C2—H2A	119.6	C23—C24—H24A	120.5
C3—C2—H2A	119.6	C24—C25—C26	120.88 (15)
N2—C3—C2	122.06 (13)	C24—C25—H25A	119.6
N2—C3—C4	121.03 (12)	C26—C25—H25A	119.6
C2—C3—C4	116.88 (12)	C21—C26—C25	119.95 (13)
C5—C4—C3	120.87 (12)	C21—C26—H26A	120.0
C5—C4—H4A	119.6	C25—C26—H26A	120.0
C3—C4—H4A	119.6	N1—C27—C28	113.66 (13)
C4—C5—C6	122.52 (12)	N1—C27—H27A	108.8
C4—C5—H5A	118.7	C28—C27—H27A	108.8
C6—C5—H5A	118.7	N1—C27—H27B	108.8
C1—C6—C5	116.11 (12)	C28—C27—H27B	108.8
C1—C6—C7	122.20 (11)	H27A—C27—H27B	107.7
C5—C6—C7	121.69 (11)	C27—C28—H28A	109.5
N3—C7—C6	110.63 (10)	C27—C28—H28B	109.5
N3—C7—C8	112.90 (10)	H28A—C28—H28B	109.5
C6—C7—C8	110.18 (10)	C27—C28—H28C	109.5
N3—C7—C14	99.96 (10)	H28A—C28—H28C	109.5
C6—C7—C14	113.21 (10)	H28B—C28—H28C	109.5
C8—C7—C14	109.67 (10)	N1—C29—C30	113.30 (15)
C13—C8—C9	115.92 (11)	N1—C29—H29A	108.9
C13—C8—C7	122.18 (12)	C30—C29—H29A	108.9
C9—C8—C7	121.79 (11)	N1—C29—H29B	108.9
C10—C9—C8	123.15 (12)	C30—C29—H29B	108.9
C10—C9—H9A	118.4	H29A—C29—H29B	107.7
C8—C9—H9A	118.4	C29—C30—H30A	109.5
C9—C10—C11	120.28 (12)	C29—C30—H30B	109.5
C9—C10—H10A	119.9	H30A—C30—H30B	109.5
C11—C10—H10A	119.9	C29—C30—H30C	109.5
N1—C11—C12	121.39 (12)	H30A—C30—H30C	109.5
N1—C11—C10	121.54 (12)	H30B—C30—H30C	109.5
C12—C11—C10	117.07 (11)	N2—C31—C32	114.59 (13)
C13—C12—C11	120.85 (12)	N2—C31—H31A	108.6
C13—C12—H12A	119.6	C32—C31—H31A	108.6
C11—C12—H12A	119.6	N2—C31—H31B	108.6
O1—C13—C8	122.99 (11)	C32—C31—H31B	108.6
O1—C13—C12	114.28 (11)	H31A—C31—H31B	107.6
C8—C13—C12	122.73 (12)	C31—C32—H32A	109.5
C19—C14—C15	120.55 (12)	C31—C32—H32B	109.5
C19—C14—C7	110.74 (11)	H32A—C32—H32B	109.5
C15—C14—C7	128.69 (12)	C31—C32—H32C	109.5
C14—C15—C16	117.98 (14)	H32A—C32—H32C	109.5
C14—C15—H15A	121.0	H32B—C32—H32C	109.5
C16—C15—H15A	121.0	N2—C33—C34	113.87 (12)
C15—C16—C17	121.11 (14)	N2—C33—H33A	108.8
C15—C16—H16A	119.4	C34—C33—H33A	108.8
C17—C16—H16A	119.4	N2—C33—H33B	108.8
C18—C17—C16	120.57 (13)	C34—C33—H33B	108.8
C18—C17—H17A	119.7	H33A—C33—H33B	107.7
C16—C17—H17A	119.7	C33—C34—H34A	109.5
C17—C18—C19	117.97 (14)	C33—C34—H34B	109.5
C17—C18—H18A	121.0	H34A—C34—H34B	109.5
C19—C18—H18A	121.0	C33—C34—H34C	109.5
C14—C19—C18	121.81 (13)	H34A—C34—H34C	109.5
C14—C19—C20	109.47 (11)	H34B—C34—H34C	109.5
C18—C19—C20	128.71 (13)	C11—N1—C27	121.45 (12)
O2—C20—N3	126.65 (13)	C11—N1—C29	121.98 (12)
O2—C20—C19	127.22 (13)	C27—N1—C29	116.38 (11)
N3—C20—C19	106.13 (11)	C3—N2—C33	120.59 (12)
C26—C21—C22	119.45 (13)	C3—N2—C31	121.56 (12)
C26—C21—N3	120.83 (12)	C33—N2—C31	117.48 (12)
C22—C21—N3	119.70 (12)	C20—N3—C21	122.99 (11)
C23—C22—C21	119.97 (14)	C20—N3—C7	113.63 (10)
C23—C22—H22A	120.0	C21—N3—C7	122.30 (10)
C21—C22—H22A	120.0	C1—O1—C13	118.40 (10)
C24—C23—C22	120.65 (14)		

### Hydrogen-bond geometry (Å, °)

**Table:**

*D*—H···*A*	*D*—H	H···*A*	*D*···*A*	*D*—H···*A*
C31—H31B···O2^i^	0.99	2.56	3.4032 (19)	144

Symmetry codes: (i) −*x*+3/2, *y*+1/2, −*z*+1/2.
